# Microstructural white matter alterations associated with migraine headaches: a systematic review of diffusion tensor imaging studies

**DOI:** 10.1007/s11682-022-00690-1

**Published:** 2022-06-16

**Authors:** Rahil Rahimi, Mahsa Dolatshahi, Fatemeh Abbasi-Feijani, Sara Momtazmanesh, Giulia Cattarinussi, Mohammad Hadi Aarabi, Lorenzo Pini

**Affiliations:** 1 Faculty of Medicine, Tehran University of Medical Sciences, Tehran, Iran; 2 School of Medicine, Arak University of Medical Sciences, Tehran, Iran; 3 Department of Neuroscience and Padova Neuroscience Center (PNC), University of Padova, Padua, Italy

**Keywords:** Migraine, Diffusion tensor imaging, Tractography, White matter, Fractional anisotropy

## Abstract

The pathophysiology of migraine as a headache disorder is still undetermined. Diffusion tensor imaging (DTI) has significantly improved our knowledge about brain microstructure in this disease. Here, we aimed to systematically review DTI studies in migraine and survey the sources of heterogeneity by investigating diffusion parameter changes associated with clinical characteristics and migraine subtypes. Microstructural changes, as revealed by widespread alteration of diffusion metrics in white matter (WM) tracts, subcortical and cortical regions, were reported by several migraine DTI studies. Specifically, we reported changes in the corpus callosum, thalamic radiations, corona radiata, and brain stem. These alterations showed high variability across migraine cycle phases. Additionally, migraine associated with depressive/anxiety symptoms revealed significant changes in the corpus callosum, internal capsule, and superior longitudinal fasciculus. No significant WM microstructural differences were observed between migraine patients with and without aura. Overall, differences between chronic and episodic migraine showed inconsistency across studies. Migraine is associated with microstructural changes in widespread regions including thalamic radiations, corpus callosum, and brain stem. These alterations can highlight neuronal damage and neuronal plasticity mechanisms either following pain stimulations occurring in migraine cycle or as a compensatory response to pain in chronic migraine. Longitudinal studies applying advanced modalities may shed new light on the underlying microstructural changes in migraine subtypes.

## Introduction

Migraine is a major neurological disorder characterized by moderate or severe headaches with a unilateral, pulsatile quality accompanied by a myriad of symptoms, such as nausea, photophobia, and phonophobia. There are several categorizations for migraine, such as chronic (CM) and episodic migraine (EM), migraine with aura (MWA), and migraine without aura (MWoA), i.e., the presence/absence of sensory disturbances, including flashes of light, blind spots, and hand/face tingling, respectively ("Headache Classification Committee of the International Headache Society (IHS) The International Classification of Headache Disorders, 3rd edition," 2018). According to the global burden of disease study in 2017, headache disorders were the second most prevalent disease and the second-highest contributor to age-standardized global years lost due to disability (YLD). Among headache disorders, migraine was in the first rank based on YLD and in the second rank based on prevalence (Sevenich, 2018). Despite the high prevalence and the well-known clinical features of this disease, the cascade of events triggering initiation and disease progression is far from being understood.

Recently, it has been suggested that alterations in mechanisms involved in cortical excitatory-inhibitory balance may promote hyper-reactivity to pain in individuals genetically susceptible to migraine (Gasparini et al., 2013; Mainero et al., 2011). Because of this increased neuronal excitation, a wave of cortical spreading depression (CSD) turns up and spreads across the cerebral cortex (Charles & Baca, 2013; Granziera et al., 2006). Animal studies showed that neuronal excitation and the subsequent CSD might lead to several alterations, such as activation of trigeminal afferent neurons (Karatas et al., 2013; Moskowitz et al., 1993), increasing brain vascular permeability, and promoting neuroinflammation (Cutrer et al., 2012; Gursoy-Ozdemir et al., 2004). The latter can trigger neuronal firing in the spinal and trigeminal nucleus and in the meningeal nociceptors (Kincses et al., 2019). Thus, the recurrent sensitization of the trigeminovascular system linked with the increased reactivity to stimuli is considered the main trigger of the cascade of events associated with migraine attacks (DaSilva et al., 2007; Welch, 2005).

The trigeminal complex projects to the brainstem, hypothalamus, basal ganglia, thalamic, and cortical regions involved in sensory and cognitive pain input and aura (Dodick, 2018; Noseda et al., 2011). Projection neurons can modulate central signals from the periaqueductal gray matter (PAG), dorsolateral pons, medullary raphe, spinal trigeminal nucleus (SpV), as well as the descending cortical inhibitory complex (Marciszewski et al., 2018). These areas mediate the intensity of sensory stimuli, cerebral blood flow, and nociception of cortical and subcortical neurons (Dodick, 2018; Maniyar et al., 2014) exhibiting different levels of activity in the migraine stages (Dodick, 2018; Moulton et al., 2008). Similarly, the thalamus, a bilateral brain structure projecting out to the cerebral cortex through several tracts, such as the fornix, cingulum, anterior thalamic radiations (ATR) and posterior thalamic radiations (PTR) (Jones, 2002; Zhang et al., 2010), is involved in the pathophysiology of migraine (Li et al., 2011; Yuan et al., 2012).

Despite these significant advancements in the comprehension of the potential neural mechanisms linked with migraine, we still lack effective imaging biomarkers aimed at predicting the disease course or treatment response (Dodick, 2018). In this regard, the analysis of the human connectome, i.e., the human brain organization into highly interconnected regions (Sporns et al., 2005), could help identify new non-invasive cost-effective biomarkers for diagnosis, progression, and as surrogate outcomes for clinical trials (Dodick, 2018; Katsarava et al., 2012). In the last years, diffusion-weighted imaging (DWI) sequences, based on the difference in magnitude of water diffusion, have improved our knowledge of neurological disorders. Diffusion tensor imaging (DTI) comprises a group of techniques computing eigenvalues (λ1, λ2, and λ3) and eigenvectors (ε1, ε2, and ε3) used to define an ellipsoid that represents an isosurface of diffusion probability aimed at understanding the microstructural properties of the brain tissue (Huisman, 2010; O'Donnell & Westin, 2011; Zhang et al., 2020). Four DTI indices are commonly used to quantify the shape of the tensors in each brain voxel. The fractional anisotropy (FA) is the most widely used anisotropy measure, an index of the amount of diffusion asymmetry within a voxel. When λ1 = λ2 = λ3, the diffusion ellipsoid is a sphere indicating a perfect isotropic diffusion (FA = 0). With progressive diffusion anisotropy, the eigenvalues become more unequal, and FA values became higher. A complementary measure to FA is mean diffusivity (MD) computed as the average of the three eigenvalues of the tensor. Finally, AD and RD could be helpful in determining the diffusivity direction, along the main axis (λ1) or perpendicular to it (average of λ2 and λ3). FA is sensitive to axonal integrity, although many factors are linked with FA changes (i.e., cell death, gliosis, demyelination, increase in extracellular or intracellular liquid content, inflammation, and axonal loss). Therefore, FA is not a specific parameter to define the type of changes (Neeb et al., 2015; O'Donnell & Westin, 2011; Zhang et al., 2020) and is usually paired with MD. High MD indicates increased extracellular spaces because of shrinkage or degeneration of axons and dendritic fibers. Thus, MD is higher in cerebrospinal fluid (CSF) compared to GM and WM, as water molecules can move freely (Narr et al., 2009; Tromp & Scalars, 2016). Finally, AD and RD could be used to detect axon myelination or pathology (Zhang et al., 2020). AD is sensitive to axonal degeneration, which is associated with fiber density and axon intrinsic characteristics (Messina et al., 2015; Neeb et al., 2015). Whereas demyelination, abnormal axonal diameter, or density may influence RD (Messina et al., 2015; Tromp & Scalars, 2016).

According to a recent coordinate-based meta-analysis consisting of both volume and surface GM and DTI studies, there is no clear consensus about brain structural alterations in migraine (Masson et al., 2021a, b). However, several DTI studies showed widespread alterations of the diffusivity metrics, suggesting a multifaceted association between migraine and brain structural connection/organization (Kim et al., 2021). Herein, we aim to systematically review DTI studies and comprehensively discuss microstructural changes in migraine. Moreover, we aim to clarify whether these changes are associated with clinical parameters, including attack duration, frequency, disease duration, and different phases of migraine.

## Methods

This systematic review was performed in accordance with the Preferred Reporting Items for Systematic Reviews and Meta-Analyses (PRISMA) guidelines (Moher et al., 2009).

### Literature search and selection criteria

We performed an online search in PubMed and Scopus databases in January 2022. The search terms included "Diffusion Tensor Imaging OR Diffusion Magnetic Resonance Imaging OR Diffusion-Weighted Imaging OR Fractional Anisotropy OR Diffusivity OR Tractography" AND "Migraine OR Migraine Disorders OR Migraine Headaches OR Migraine with Aura OR Migraine without Aura OR Chronic Migraine OR Episodic Migraine", and the equivalent search terms in each database. Reference lists of the included studies and other relevant studies were also reviewed for eligible studies.

Original studies in English were included if they (1) measured tract-based or region of interest (ROI) diffusion metrics through computational DTI methods and (2) compared microstructural changes in patients with migraine with healthy controls (HCs) or microstructural features between patients with different migraine types (e.g., with or without aura).

We excluded (1) case reports, case series, letters, commentaries, abstracts, review articles, and animal or in vitro studies, (2) studies including patients diagnosed with different neurologic conditions, and (3) interventional studies.

Data selection was performed in concordance with the PRISMA guidelines (Moher et al., 2009). Two authors (RR and MHA) independently assessed the eligibility criteria of the studies. In case of conflicting judgments, a third author’s (MD) opinion was asked.

### Data extraction

The extracted data included: (1) demographic features of the samples, including age and sex of patients and HCs, (2) data related to the disease characteristics, including classification of migraine (with or without aura), disease duration, and attack frequency and duration, (3) the characteristics of image acquisition, including field strength and b-value, (4) DTI analysis methodology, (5) the spectrum of data analysis (whole brain or tract-based), (6) key findings, including the alterations of diffusion metrics across brain regions or tracts, and (7) other relevant findings.

## Results

### Study selection

**Fig. 1:**
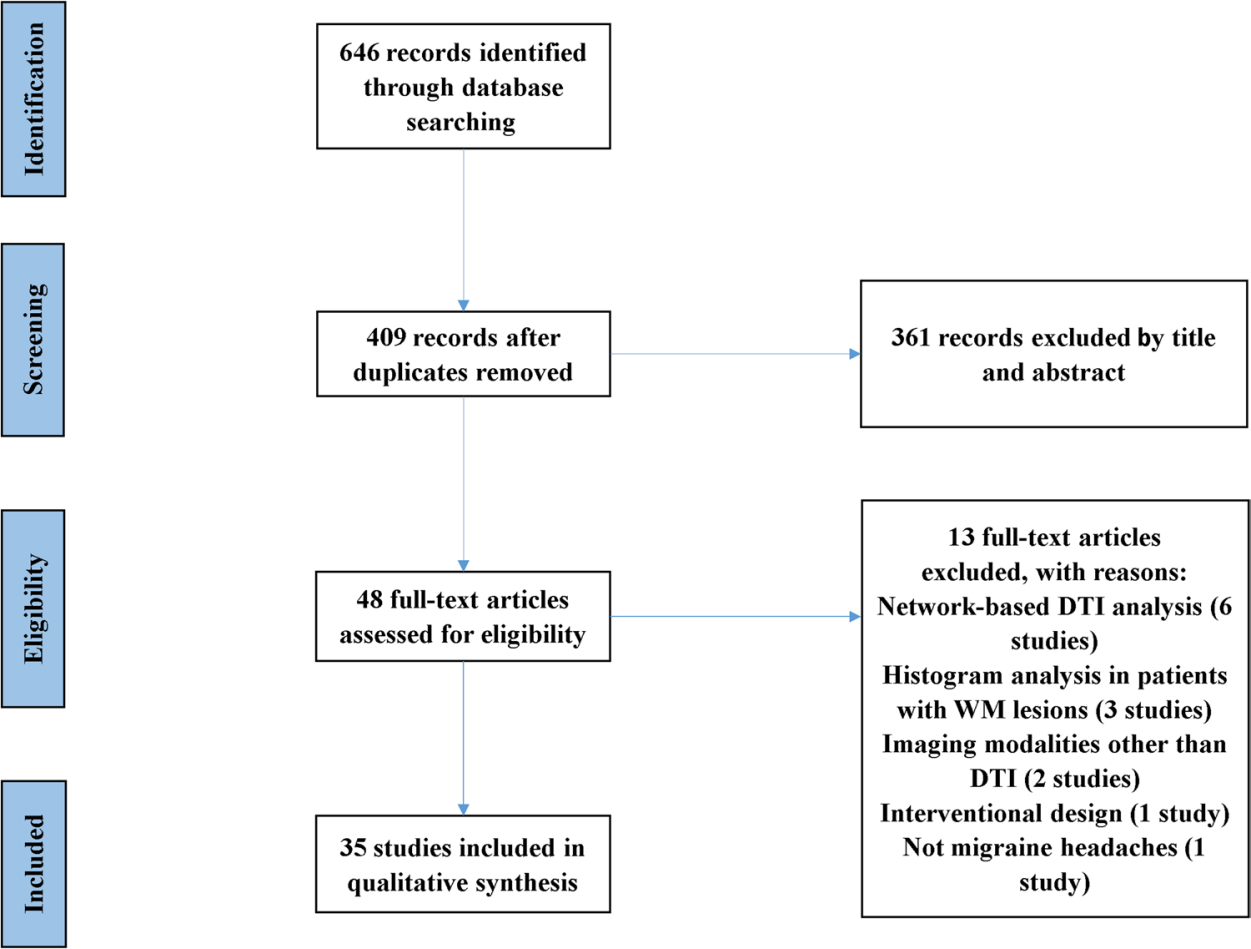
PRISMA flow diagram for the systematic review of diffusion tensor imaging studies in migraine headache patients

The PRISMA chart for studies selection is depicted in Fig. 1. A total of 646 articles were identified. After removing duplicate records, title and abstract of 409 studies were screened, leading to the exclusion of 361 studies. Of the remaining 48 studies that entered full-text screening, 35 studies were finally included. Thirteen studies were excluded due to the following reasons: network-based DTI analysis (six studies), histogram analysis in patients with WM lesions (three studies), using of imaging modalities other than DTI (two studies), interventional design (one study), and participants with a different neurological condition (one study). Table 1 summarizes the included studies. Reviewed articles included data from 2220 individuals (574 males) consisting of 1253 individuals diagnosed with migraine (295 male patients) by the International Classification of Headache (ICHD) criteria (second and third edition) (Headache Classification Committee of the International Headache, 2013; "Headache Classification Committee of the International Headache Society (IHS) The International Classification of Headache Disorders, 3rd edition," 2018; Olesen & Steiner, 2004), and 967 HCs.

**Table 1:** Diffusion imaging studies included in the review

Study	Subgroups	Patients (male)	Controls (male)	Age patients	Age controls	Disease duration (years)	Attack frequency^*^	Attack duration	Average Pain intensity
Granziera et al., 2006	MWA / MWoA	12 (3) / 12 (5)	15 (4)	33.8 ± 9.1 / 35.2 ± 6.2	33.2 ± 8.7	20.3 ± 11.2 / 19.5 ± 8.5	4.1 ± 3.6 / 4.0 ± 3.6 m^**^		
DaSilva et al., 2007	MWA / MWoA	12 (3) / 12 (5)	12 (3)	33.8 ± 9.2 / 36.0 ± 7.7	31.0 ± 8.0	20.3 ± 11.2 / 19.5 ± 8.5	4.1 ± 3.6 / 4.0 ± 3.6 m		
Schmitz et al., 2008	MWA / MWoA	8 (0) / 20 (0)	28 (0)	41.25 ± 14.51 / 44.03 ± 9.43	42.5 ± 9.31	25.75 ± 14.49 / 33.5 ± 8.98	3.44 ± 2.13 / 3.60 ± 2.00 m		
Rocca et al., 2008	MWA / MWoA	7 / 8 (1)	11(1)	43.3	41.6	25.6	21.0 y		
Li et al., 2011	MWoA / C-MWoA	12 (6) / 12(4)	12(5)	38.33 ± 8.69 / 40.42 ± 10.77	38.83 ± 8.11	12.00 ± 5.95 / 20.92 ± 9.90	5.00 ± 1.19 / 16.33 ± 4.47 m		7.75 ± 1.29 / 7.33 ± 1.72
Szabo et al., 2012	MWA / MWoA	3(0) / 18(0)	17 (0)	34.65 ± 10.86	33.27 ± 11.32	17.41 ± 9.59	33.1 ± 15.64 y***		
Kara et al., 2013	MWoA	14 (2)	15 (7)	36.4	25.9	12.5	5.2 m		
Yuan et al., 2012	MWoA	21(5)	21 (5)	32.4 ± 10.3	31.6 ± 9.5	10.6 ± 6.6	4.5 ± 1.7 m	13.4 ± 5.8	5.3 ± 1.5
Yu et al., 2013a, b	MWoA	20 (4)	20 (4)	36.1 ± 10.2	31.5 ± 13.9	11.1 ± 6.4	5.3 ± 3.7 m	15.9 ± 14.0	5.8 ± 1.7
Yu et al., 2013a, b	SDS + / SDS- (MWoA)	20 (4) / 20 (7)	40 (10)	38.2 ± 8.4 / 33.6 ± 11.7	33.2 ± 9.5	9.2 ± 5.8 / 12.4 ± 8.0	6.3 ± 4.1 / 4.2 ± 2.7 m		5.5 ± 1.9 / 4.8 ± 1.4
Liu et al., 2013	EMWoA (1 year follow-up)	21	21	22.0 ± 2.0	22.0 ± 2.0	8.2 ± 5.9 / 6.7 ± 3.3	4.4 ± 2.3 / 9.5 ± 4.7 m		5.6 ± 0.8 / 5.3 ± 1.4
Coppola et al., 2014	MWoA MO / MI	14 (3) / 10 (2)	15 (4)	31.6 ± 7.6 / 33.3 ± 12.1	28.6 ± 4.0	16.5 ± 6.6 / 13.1 ± 9.9	3.4 ± 2.4 / 4.0 ± 3.4 m	18.8 ± 20.5 / 39.6 ± 24.6	7.5 ± 0.8 / 7.4 ± 0.6
Neeb et al., 2015	EMWoA / CMWoA	21 (6) / 21 (6)	21 (6)	49.36 ± 7.62 / 49.04 ± 7.46	49.40 ± 7.79	26.71 ± 14.42 / 24.43 ± 8.3	5.33 ± 1.59 / 17.38 ± 2.66 m		6.19 ± 1.4 / 7.05 ± 1.63
Tessitore et al., 2015	MWA / MWoA	20 (8) / 20(8)	20 (8)	30.10 ± 1.66 / 30.05 ± 1.53	29.15 ± 1.30	10.96 ± 1.91 / 11.20 ± 1.75	8.85 ± 1.70 / 79.80 ± 8.67 days/y		7.96 ± 0.23 / 8.57 ± 0.24
Chong & Schwedt 2015	MWoA / MWA	23 (8) 10 / 13)	18 (6)	38.5 ± 11.0	37.7 ± 11.0	18.0 ± 11.0	7.9 ± 5.1 m		
Messina et al., 2015	EMWA / EMWoA	8 (3) / 7 (4)	15 (7)	14.9 ± 1.5 / 13.1 ± 3.5	13.8 ± 2.7	3.3 ± 2.3 / 2.8 ± 3.3	11 ± 12.3 / 33 ± 12.4 y		
Tedeschi et al., 2016	EMWA / EMWoA	20(8) / 20(8)	20(8)	30.10 ± 7.4 /30.05 ± 6.9	29.15 ± 5.8	10.96 ± 8.6 / 11.20 ± 7.8	8.85 ± 7.6 / 79.80 ± 38.8 days/y		7.96 ± 1.1 / 8.57 ± 1.03
Gomez-Beldarrain et al., 2015	EM / CM	19 (0) / 18 (4)	15 (1)	41.37 ± 7.86 / 43.78 ± 7.91	45.73 ± 6.78				
Delic et al., 2016	mBTI with PTM / mBTI without PTM / migraine control	57 (39)/ 17 (12) / 20 (10)	22 (10)	17.6 / 19.7 / 21.7	18.8				
Coppola et al., 2016a, b	MWoA	13 (3)	19 (7)	34.4 ± 10.7	31.7 ± 4.0	15.0 ± 9.6	3.9 ± 3.1 m	43.4 ± 28.4	7.4 ± 0.7
Coppola et al., 2016a, b	EMWoA	19 (7)	18 (6)	29.7 ± 4.0	32.4 ± 7.2	14.1 ± 6.8	3.1 ± 2.1 m	27.3 ± 30.0	7.3 ± 0.9
Zhang et al., 2017	EMWoA	32 (8)	32 (8)	38.3 ± 10.16	38.8 ± 10.02	9.5 ± 6.23	3.36 ± 2.55 m	20.5 ± 20.02	7.3 ± 2.04
Szabo et al., 2018	MWA/MWoA	18 (3) / 25 (3)	28 (3)	32.11 ± 8.01 / 35.69 ± 8.61	31.74 ± 9.58	14.89 ± 8.45 / 12.76 ± 9.97	29.03 ± 25.31 / 46.22 ± 33.48 y		
Marciszewski et al., 2018	pooled MWA/MWoA group	25 (4)	57 (14)	30.2 ± 2.0	28.3 ± 1.3	14.6 ± 2.2	18.9 ± 2.3 y		3.7 ± 0.1 6-point VAS (0 to 5 score)
Petrušić et al., 2018	MVA + / MVA	22 (4)/ 21 (8)	20 (4)	38.7 ± 13.3 / 39.0 ± 9.0	41.6 ± 12.5	18.8 ± 11.2 /17.1 ± 10.2	6.3 ± 6.1 / 5.9 ± 4.8 y		
Shibata et al., 2018	MWoAD + / MWoAD- / MWAD + / MWAD-	13 (0) / 68 (9) / 5 (0) / 17 (3)	46 (9)	43.4 ± 15.7 / 40.8 ± 16.5 / 38.0 ± 4.5 / 40.1 ± 17.8	38.4 ± 12.7	21.6 ± 5.0 / 18.1 ± 1.8 / 20.4 ± 4.3 / 13.9 ± 2.6	8.6 ± 1.8 / 5.0 ± 0.6 / 8.0 ± 2.1 / 3.3 ± 0.6 m		
Chong et al., 2019	Migraine patients / PPTH	41 (15) / 46 (32)	41 (22)	39.1 ± 10.7 / 37.7 ± 10.6	38.1 ± 10.2	22.2 ± 13.9 / 10.0 ± 8.1	16.6 ± 8.4 / 16.1 ± 8.5 m		
Marciszewski et al., 2019	pooled MWA/MWoA group	36 (8)	29 (5)	30.6 ± 1.7	31.9 ± 2.3	16.2 ± 1.9	1.3 ± 0.1 m		3.8 ± 0.1 6-point VAS (0 to 5 score)
Kattem Husøy et al., 2019	Migraine	69 (16)	277 (168)	57.5 ± 4.3	58.7 ± 4.1				
Qin et al., 2019	MWoA	46 (15)	46 (15)	38.87 ± 11.6	39.0 ± 11.2	8.6 ± 6.2	3.3 ± 2.8 m	17.3 ± 19.5	7.1 ± 1.9
Planchuelo-Gómez et al., 2020	EM / CM	54 (9) / 56 (6)	50 (11)	37.1 ± 8.2 / 38.1 ± 8.7	36.1 ± 13.2	14.1 ± 11.1 / 19.6 ± 10.4	3.6 ± 1.9 / 13.9 ± 6.9 days/m		
Russo et al., 2020	MWoA dCA / MWoA ndCA	20 (4) / 17(7)	19(6)	31.3 ± 1.97 / 30.7 ± 2.15	28.8 ± 1.45	13.8 ± 1.77 / 12.4 ± 2.38)	T0 (4.4 ± 0.94 / 3.3 ± 0.57) days/m / T1 (4.7 ± 1.01 / 3.5 ± 0.71) days/m		T0 (8.2 ± 0.31 / 7.9 ± 0.36)/ T1 (8.4 ± 0.26 / 7.9 ± 0.34)
Coppola et al., 2020	EMWoA / CMWoA	19 (6) / 18 (7)	18 (7)	31.9 ± 2.1 / 31.5 ± 10.1	28.4 ± 4.1	14.2 ± 6.7 / 14.8 ± 12.8	3.2 ± 2.1 number per m / 23.0 ± 7.3 days/m		7.5 ± 0.8/ 7.4 ± 0.6
Masson et al., 2021a, b	MWoA	19 (6)	19 (6)	32.7 ± 8.7	33.6 ± 11.5	16.8 (7.4)	3.3 (1.1) m		
Pak et al., 2021	MOH + / MOH -	25 (3) / 33(6)		36.80 ± 1.60 / 35.36 ± 1.37		6.68 ± 1.07 / 7.87 ± 1.47	11.48 ± 0.88 / 5.60 ± 0.62 m		9.20 ± 0.17 / 8.63 ± 0.24

*Data are reported as mean and standard deviation.* *CMWA* chronic migraine with aura; *CMWoA* chronic migraine without aura; *MWoAD* migraine without aura plus depressive/anxious disorder; *EMWA* episodic migraine with aura; *EMWoA* episodic migraine without aura; *MI* migraine during attack (ictal); *MO* migraine between attacks (interictal period); *MOH +*  Medication overuse headache; *MOH-* did not overuse medication; *mTBI* mild traumatic brain injury; *MVA +*  migraine with visual aura; *MVA-* migraine without visual aura; *MWA* migraine with aura; *MWoA* migraine without aura; *MWoAD +*  MWoA with medication overuse headache; *MWOAD-* MWoA without medication overuse headache; *MWAD +* MWA with medication overuse headache; *MWAD-* MWA without medication overuse headache; *ndCA* non-developing cutaneous allodynia; *PPTH* persistent post-traumatic headache; *PTM* posttraumatic migraines; *SDS +*  self-rating depression scale > 49, *SDS-* self-rating depression scale <  = 49; *Simple MWoA* migraine without aura and without depressive/anxious disorder; *VAS* visual analogue scale. *: number of migraine frequencies; **: per month; ***: per year

### Study characteristics

Data for mean disease duration (years since patients received migraine diagnosis) and attack frequency (the number of migraine headache attacks per month or days) were reported for all articles but Delic et al. (2016), Gomez-Beldarrain et al. (2015), and Kattem Husoy et al. (2019). Mean disease duration ranged from less than three years (Messina et al., 2015) to about 34 years (Schmitz et al., 2008) among the included articles. Several studies reported mean attack duration, defined as the number of hours each attack lasts (Coppola et al., 2014, 2020; Liu et al., 2013), and pain intensity (Coppola et al., 2016a, b; Coppola et al., 2020; Coppola et al., 2014; Li et al., 2011; Liu et al., 2013; Marciszewski & Meylakh, 2019; Marciszewski et al., 2018; Neeb et al., 2015; Russo et al., 2020; Tantik Pak et al., 2021; Tedeschi et al., 2016; Tessitore et al., 2015; Yu et al., 2013b; Zhang et al., 2017), based on a pain scale continuum ranging from 0 (no pain) to 10 (severe pain).

Among the included studies, eight studies compared MWoA to MWA, and four studies compared CM to EM. In 29 of the included studies, HCs were matched by sex and age. Patients and HCs were matched for hand predominance in 15 studies to attenuate possible confounding effects. In 10 studies, patients were under pharmacological treatment. In addition, 15 studies applied questionnaires to investigate depression, anxiety, quality of life, and disability in their cohorts.

Most of the studies investigated microstructural metrics at whole brain level. Nine studies focused on specific pathways as regions of interest: optic radiation (OR) (Rocca et al., 2008), corticospinal tract (CST) (Rocca et al., 2008), corpus callosum (CC) (Li et al., 2011; Rocca et al., 2008), red nuclei, PAG, thalami, posterior limbs of IC (PLICs), subcortical WM (Kara et al., 2013), thalamus (Coppola et al., 2016a, b; Coppola et al., 2014), cerebellum and brainstem (Qin et al., 2019), anterior insula, cingulate gyrus, uncinate fasciculus (Gomez-Beldarrain et al., 2015), and orbitofrontal cortex (Tantik Pak et al., 2021). Table 2 summarizes the imaging methodologies of the included studies.

**Table 2:** Diffusion Imaging methodology of the studies included in the review

Study		Field strength	b values	DTI analysis method	Directions	Regions/tracts studied
1	Granziera et al., 2006	3	700	ROI, VBA	60 directions	whole brain
2	DaSilva et al., 2007	3	700	ROI	60 directions	whole brain
3	Schmitz et al., 2008	3	1000	VBM, Modified optimized VBM procedure	6 orthogonal diffusion gradients	whole brain
4	Rocca et al., 2008	3	1000	Tractography, VBA	32 non-collinear directions	optic radiation, corticospinal tract and the corpus callosum (as control)
5	Li et al., 2011	3	1000	ROI, VBA	12 directions	corpus callosum
6	Szabo et al., 2012	1.5	1000	TBSS	60 directions	whole brain
7	Kara et al., 2013	3	1000	ROI	55 directions	red nuclei, PAG, thalami, PLIC, and SWM
8	Yuan et al., 2012	3	1000	TBSS	30 non-linear directions	whole brain
9	Yu et al., 2013a, b	3	1000	TBSS	30 non-linear directions	whole brain
10	Yu et al., 2013a, b	3	1000	TBSS	30 noncollinear directions	whole brain
11	Liu et al., 2013	3	1000	ROI	30 non-linear directions	whole brain
12	Coppola et al., 2014	3	1000	ROI	30 non-collinear directions	right and left thalamus
13	Neeb et al., 2015	3	1000	TBSS	67 diffusion directions	whole brain
14	Tessitore et al., 2015	3	1000	TBSS, VBM	32 isotropically distributed gradients	whole brain
15	Chong & Schwedt 2015	3	1000	Global tractography	30-non-linear directions	18 major fiber tract bundles: bilateral ATR, cingulum angular bundles, cingulum cingulate gyri, CST, SLF (parietal, temporal), ILF, uncinate fasciculi, forceps major/minor tracts
16	Messina et al., 2015	3	900	TBSS, DT probabilistic tractography	35 non-collinear directions	whole brain
17	Tedeschi et al., 2016	3	1000	TBSS, VBM	32 isotropically distributed gradients	whole brain
18	Gomez-Beldarrain et al., 2015	3	800	TBSS (ROI)	15 spherically distributed axes	Anterior insula, Cingulate gyri, Uncinate fasciculus
19	Delic et al., 2016	1.5	1000	Histogram analysis	25 non-collinear directions	whole brain
20	Coppola et al., 2016a, b	3	1000	VBM, ROI	30 non-collinear directions	Bilateral thalami
21	Coppola et al., 2016a, b	3	1000	ROI	30 non-collinear directions	Bilateral thalami
22	Zhang et al., 2017	3	1000	VBM, SBM, DTI analysis	64 different directions	whole brain
23	Szabo et al., 2018	1.5	1000	TBSS	60 directions	whole brain
24	Marciszewski et al., 2018	3	1000	VBM, VBA	32 independent orientations	whole brain
25	Petrušić et al., 2018	1.5	1000	TBSS, Probabilistic tractography analysis	60 mutually non-parallel directions	whole brain
26	Shibata et al., 2018	1.5	1000	TBSS, ROI	6 different directions	whole brain
27	Chong et al., 2019	3	1000	Tractography	30 non-linear directions	whole brain
28	Marciszewski et al., 2019	3	1000	VBA	32 independent orientations	whole brain
29	Kattem Husøy et al., 2019	1.5	1000	TBSS, Tractography	40 non-colinear directions	whole brain
30	Qin et al., 2019	3	1000	VBM/ cerebellar tract analysis	64 directions	cerebellum and brainstem
31	Planchuelo-Gómez et al., 2020	3	1000	TBSS	61 gradient directions	Whole brain
32	Russo et al., 2020	3	1000	TBSS, VBM	NM	whole brain
33	Coppola et al., 2020	3	1000	TBSS	30 diffusion directions	whole brain
34	Masson et al., 2021a, b	3	1000	TBSS, VBM, SBM	64 gradient directions	whole brain
35	Tantik Pak et al., 2021	1.5	1000	ROI	24 diffusion-encoding directions	orbitofrontal cortex

*ADC* apparent diffusion coefficient; *ATR* anterior thalamic radiation; *CST* corticospinal tract; *DTI* diffusion tensor imaging; *ILF* inferior longitudinal fasciculus; *PAG* periaqueductal gray matter; *PLICs* posterior limbs of internal capsules; *ROI* region of interest; *SBM* surface-based morphometry; *SLF* superior longitudinal fasciculus; *SWM* subcortical white matter; *TBSS* tract-based spatial statistics; *VBA* voxel-based analysis; *VBM* voxel-based morphometry

### Diffusion alterations in migraine patients vs. controls

**Fig. 2:**
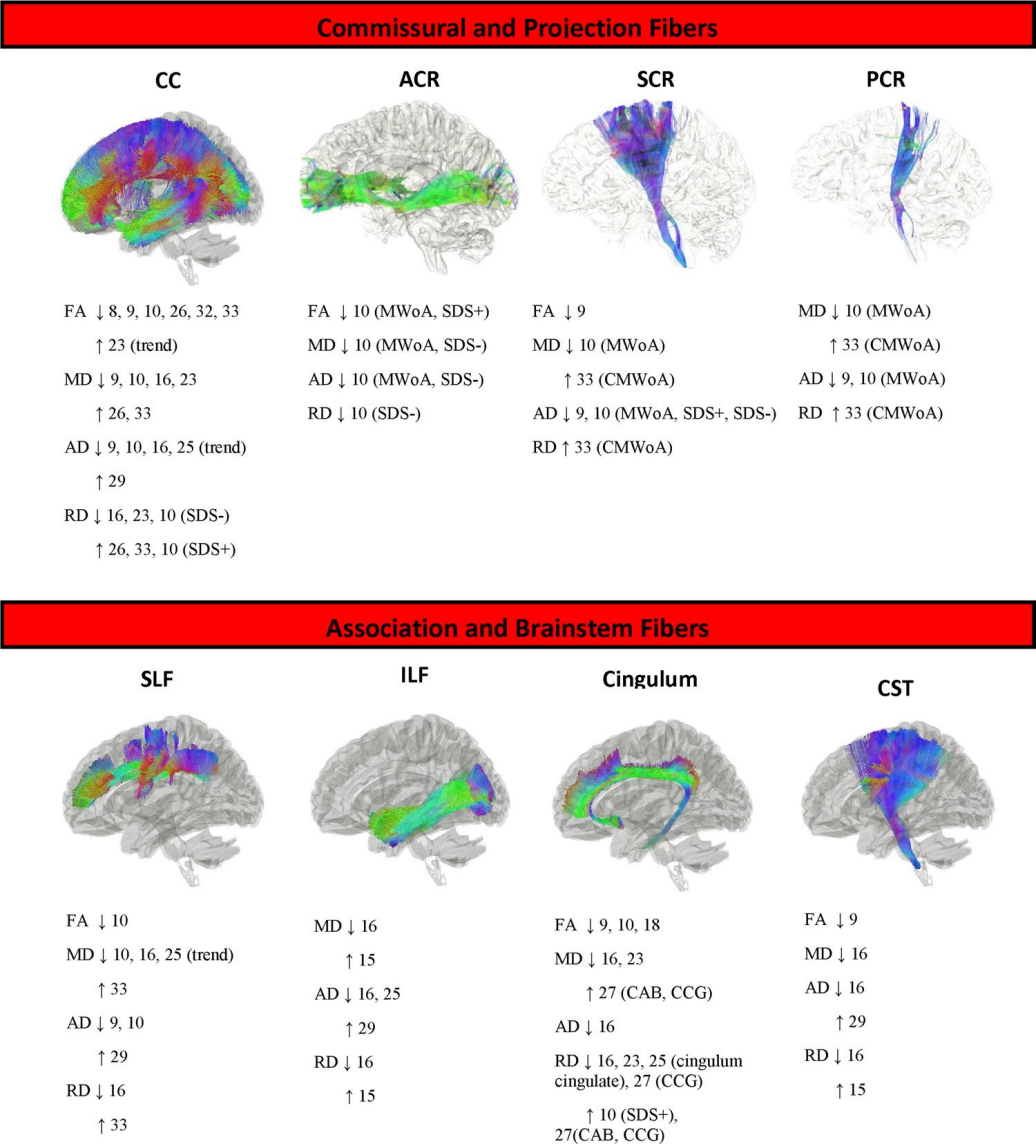
Schematic view of white matter tract changes of association, commisural, projection, and brainstem pathways in patients with migraine headache compared to healthy controls. The fiber tract visualization was adopted from the Human Connectome Project population-based atlas (available at https://brain.labsolver.org/hcp_trk_atlas.html). The study population, in which changes were observed, are reported in brackets. Upward arrrow means an increase and downward arrow means a decrease in diffusion parameters. Reference 23 reported a trend toward increased FA in CC; reference 25 reported a trends toward decreased AD in CC and decreased MD in SLF tracts in patients. Abbreviations: ACR: anterior corona radiata, AD: axial diffusivity, CAB: cingulum–angular bundle, CCG: cingulum cingulate gyrus, CC: corpos callosum, CMWoA: chronic migraine without aura, CST: corticospinal tract, FA: fractional anisotropy, ILF: inferior longitudinal fasciculus, MD: mean diffusivity, MWoA: migraine without aura, PCR: posterior corona radiata, SCR: superior corona radiata, SDS+: self-rating depression scale > 49; SDS-: self-rating depression scale < 049; RD: radial diffusivity, SLF: superior longitudinal fasciculus

The most frequently described structures exhibiting impaired integrity in migraine included brainstem, projection, association, and commissural fibers. WM tracts showing significant differences between migraine patients and controls are depicted in Fig. 2 and Table 3. Figure 2 provides a representation of WM tracts through the Human Connectome Project population-based atlas (Yeh et al., 2018) freely available online (https://brain.labsolver.org/hcp_trk_atlas.html). Migraine patients showed WM changes mainly in the CC, cingulate fibers, brainstem, thalamic radiations, and superior and inferior longitudinal fasciculus (SLF and ILF). However, several studies did not find significant alterations in FA (Chong & Schwedt, 2015; Coppola et al., 2016a; Coppola et al., 2014; Kara et al., 2013; Liu et al., 2013; Masson et al., 2021a, b; Messina et al., 2015; Neeb et al., 2015; Petrušić et al., 2018; Szabo et al., 2018; Tedeschi et al., 2016; Tessitore et al., 2015; Zhang et al., 2017), MD (Coppola et al., 2016a; Coppola et al., 2014; Liu et al., 2013; Masson et al., 2021a, b; Neeb et al., 2015; Petrušić et al., 2018; Russo et al., 2020; Szabo et al., 2018; Tedeschi et al., 2016; Tessitore et al., 2015), AD (Kara et al., 2013; Liu et al., 2013; Masson et al., 2021a, b; Neeb et al., 2015; Petrušić et al., 2018; Russo et al., 2020; Shibata et al., 2018; Szabo et al., 2018; Szabo et al., 2012; Tedeschi et al., 2016; Tessitore et al., 2015; Zhang et al., 2017) and RD (Kara et al., 2013; Liu et al., 2013; Masson et al., 2021a, b; Neeb et al., 2015; Petrušić et al., 2018; Russo et al., 2020; Szabo et al., 2012; Tedeschi et al., 2016; Tessitore et al., 2015; Yu et al., 2013a; Zhang et al., 2017).

**Table 3:** Overview of the literature (between-group differences)

Study		FA changes	MD changes	RD or AD changes	Additional imaging results
1	Granziera et al., 2006	M < HC: bilateral WM near V3A, subjacent to area MT + in the right hemisphere and superior colliculus and the left lateral geniculate nucleus MWA = MWoA			**Cortical thickness:** M > HC: Bilateral cortical thickness in V3A and MT + MWA = MWoA MWA and MWoA showed anatomical changes in cortical areas involved in motion perception
2	DaSilva et al., 2007	M < HC: PLIC at the proximity of the ventroposterior medial thalamus, CR at the level of the lateral ventricle horn along the trigeminothalamic tract M < HC: ventroposterior medial thalamus MWA < HC & MWoA: bilateral ventral trigeminothalamic tract MWoA < HC & MWA: ventrolateral periaqueductal grey matter			No associations between FA values and migraine clinical characteristics
3	Schmitz et al., 2008	M < HC: superior frontal lobe, medial frontal lobe, brainstem and cerebellum LD < SD: right frontal lobe			**ADC**: M = HC LD = SD **WM**: M < HC: frontal, occipital and parietal WM reduction M > HC: superior frontal HF < LF: right frontal lobe HF > LF: left parietal lobe LD > SD: bilateral cerebellum **GM**: M < HC: frontal lobe HF < LF: left parahippocampal, left superior frontal gyrus inferior parietal lobe, and right parahippocampal LD < SD: basal ganglia and brainstem (medulla) **CSF**: M = HC **WM lesion load**: M = HC LD = SD HF = LF
4	Rocca et al., 2008	MWA < HC: bilateral OR MWA < MWoA: right OR MWoA = HC	MWA > HC: right OR		No OR atrophy in patients with migraine No significant association between OR diffusion variables and age, disease duration, and frequency of migraine attacks
5	Li et al., 2011	Complicated MWoA < Simple MWoA < HC: genu, body, and splenium of the CC			**ADC:** No significant CC differences
6	Szabo et al., 2012	M < HC: right frontal WM	M > HC: right frontal white matter (group- level voxel-wise analysis) M = HC: No significant difference (whole brain analysis)	**AD**: M = HC **RD**: M > HC: right frontal white matter (group- level voxel-wise analysis) M = HC: (whole brain analysis)	**Tractography**: The right frontal WM fibers were connected to the ipsilateral prefrontal cortical regions, insula, thalamus, dorsal, and ventral midbrain. Fibers were running alongside the occipital cortex through the inferior fronto-occipital fasciculus. Fibers also crossed the midline through the CC No associations were found between the FA value and duration of migraine or attack frequency
7	Kara et al., 2013	M = HC: red nuclei, PAGs, thalami, PLICs and SWM regions			**ADC**: M > HC: right and left red nuclei M = HC in PAGs, thalami, PLICs and SWM regions No significant correlation between DTI indices and age, duration of disease, frequency of migraine attacks, and localization of pain
8	Yuan et al., 2012	MWoA < HC: bilateral genu and the left splenium of CC			Negative correlation between FA of the genu of CC and duration of migraine in MWoA No significant correlation between FA of the splenium of CC and duration of migraine
9	Yu et al., 2013a, b	M < HC: genu, body, and splenium part of the CC, bilateral ALICs and PLICs, bilateral cerebral peduncles, CR, cingulum bundle, CST, and thalamus	M < HC: genu, body and splenium part of the CC, right ALIC, PLICs, and thalamus radiation	**AD**: M < HC: genu, body, and splenium part of the CC, bilateral ALICs and PLICs, bilateral SCRs, bilateral SLFs and thalami **RD**: M = HC	Overlap of lower FA and AD without lower MD: genu, body, and splenium part of the CC, bilateral ALICs and PLICs, the right SCR and the left SLF Overlap of lower FA, MD, and AD: genu, body, and splenium part of the CC and the right ALIC and PLIC Overlap of lower MD, and AD without lower FA: genu, body, and splenium part of the CC, the right ALIC and PLIC, the right SCR and the left SLF Negative association between mean FA of the right PLIC, the splenium and genu of the CC and frequency of migraine attacks Negative association between mean MD and AD values of the right ALIC and splenium of the CC with the frequency of migraine attacks
10	Yu et al., 2013a, b	MWoA < HC: left ALIC, left ACR, thalamus, brainstem, left cerebral peduncle, splenium of the CC and left cingulate gyrus SDS + < HC: genu, body and splenium of the CC, left IC, left ACR, left EC, left SLF, left PTR and left cerebral peduncle SDS- = HC SDS + < SDS-: genu, body and splenium of the CC, bilateral ACR, bilateral SCR, bilateral SLF, bilateral ECs, bilateral PLIC, bilateral PTR, bilateral RIC and left ALIC	MWoA < HC: genu, body and splenium of the CC, right ALIC, right ACR, bilateral SCR, right PCR, bilateral SLF and left PTR SDS + = HC SDS- < HC: genu, body and splenium of the CC, right ACR, left PTR, bilateral SLF and bilateral SCR SDS + > SDS-: genu, body and splenium of the CC, bilateral ACR, bilateral SCR, bilateral SLF, bilateral EC, bilateral PLIC, bilateral PTR, bilateral RIC and left ALIC and right ALIC	**AD**: MWoA < HC: genu, body and splenium of the CC, bilateral ICs, bilateral ACR, right ECs, bilateral PCR, bilateral SLF, bilateral SCR, bilateral cerebral peduncles and brainstem SDS + < HC: genu, body and splenium of the CC, bilateral SCR, bilateral SLF, left cerebral peduncle, bilateral EC, bilateral IC and bilateral PTR SDS- < HC: genu, body and splenium of the CC, right ACR, right IC, right PTR, right EC and bilateral IC SDS + = SDS- **RD**: MWoA = HC SDS + > HC: increased RD in the genu and body of the CC, left cingulum and right ALIC SDS- < HC: the genu and splenium of the CC, right ACR, right SCR, right EC, right PLIC, right RIC, bilateral PTR and bilateral SLF SDS + > SDS-: increased RD in the genu, body and splenium of the CC, bilateral ACR, bilateral SCR, bilateral SLF, bilateral EC, bilateral PLIC, bilateral PTR, bilateral RIC and left ALIC and right ALIC	Overlap of SDS positive and SDS negative patients: genu, body and splenium of the CC, bilateral SCR, SLF, EC, PLIC, RIC, left PTR and right ACR Significant association between DTI indices in the right ACR, genu and splenium of the CC and left SLF and the SDS score of MWoA Significant negative association between mean FA values and the SDS scores, including right ACR, genu of the CC, splenium of the CC, and left SLF Significant positive association between mean RD values of the genu of the CC, splenium of the CC and left SLF and the SDS scores
11	Liu et al., 2013	No significant differences in newly diagnosed patients with EMWoA and after the 1-year follow-up examination (both with and without medications as covariate)	No significant differences in newly diagnosed patients with EMWoA and after the 1-year follow-up examination (both with and without medications as covariate)	No significant differences in newly diagnosed patients with EMWoA and after the 1-year follow-up examination (both with and without medications as covariate)	**GM**: widespread reduced GM at 1-year follow-up, including dorsolateral and medial part of the superior frontal gyrus, OFC, hippocampus, precuneus, inferior parietal gyrus, superior parietal gyrus, postcentral gyrus, paracentral lobule, and supramarginal gyrus
12	Coppola et al., 2014	Inter-ictal phase: MWoA > HC: bilateral thalami Ictal phase: MWoA = HC: in bilateral thalami	Inter-ictal phase: MWoA < HC in bilateral thalami Ictal phase: MWoA = MWA		**Cortical atrophy:** M = HC **WM lesions**: M = HC In migraine patients, FA of the right thalamus was positively correlated with the number of days since the last migraine attacks
13	Neeb et al., 2015	CM = EM = HC	CM = EM = HC	CM = EM = HC	**Subgroup analysis:** No significant differences in DTI stratifying for medication intake
14	Tessitore et al., 2015	MWA = MWoA = HC	MWA = MWoA = HC	MWA = MWoA = HC	**Global/local GM, WM, and CSF volumes:** MWA = MWoA = HC
15	Chong & Schwedt 2015	M = HC: bilateral ATR, bilateral CST, bilateral ILF MWA = MWoA: bilateral ATR, bilateral CST, bilateral ILF	M > HC: bilateral ATR, left CST, right ILF MWA = MWoA: bilateral ATR, bilateral CST, bilateral ILF	**RD:** M > HC: left ATR radiations, left CST, bilateral ILF MWA = MWoA: bilateral ATR, bilateral CST, bilateral ILF	Significant positive association between disease duration and MD of the right ATR and left CST
16	Messina et al., 2015	EM = HC MWA = MWoA M > HC: bilateral OR	EM < HC: bilateral optic tract, OR, CST, thalamic radiation, cingulum, ILF and IFOF, left SLF and CC MWA = MWoA M < HC: bilateral cingulum, CST, IFOF, and OR, left SLF, CC	**AD:** EM < HC: bilateral optic tract and OR, left CST, ILF and IFOF, right cingulum and CC MWA = MWoA EM < HC: bilateral cingulum, CC, Left IFOF **RD:** EM < HC: bilateral OR, trigeminothalamic tract, CST, thalamic radiation, ILF and IFOF, right cingulum and CC MWA = MWoA EM < HC: left CST, bilateral IFOF, bilateral OR, Left SLF	No correlation was reported between DTI indices with disease duration and attack frequency
17	Tedeschi et al., 2016	MWA = MWoA = HC	MWA = MWoA = HC	MWA = MWoA = HC	**Global/local GM, WM, and CSF volumes:** MWA = MWoA = HC **WM hyperintensity load**: MWoA = MWA M > HC: mild increased WM hyperintensity load
18	Gomez-Beldarrain et al., 2015	After three-months: EM = CM = HC: anterior insula, anterior cingulate gyrus, uncinate fasciculus LTCM < HC: right anterior insula, bilateral cingulate gyri, and right UF			**Preventive therapy**: Lower FA values in the left anterior insula, right cingulate gyrus and left UF **Cognitive reserve levels:** Higher cognitive: greater FA in the right anterior insula and both cingulate gyri **Anxiety levels**: Higher anxiety levels: decreased FA in the bilateral anterior insula **Physical component:** Better physical score: higher FA values in the bilateral UF
19	Delic et al., 2016	M = HC			No significant difference was seen in terms of Shannon entropy between HC and migraine patients
20	Coppola et al., 2016a, b	MWoA = HC: bilateral thalami during an attack	MWoA = HC: bilateral thalami during an attack		**Brain morphology**: MWoA = HC
21	Coppola et al., 2016a, b	MWoA > HC: bilateral thalami between attacks	MWoA < HC: tendency in the bilateral thalami between attacks		**Brain morphology**: MWoA = HC **WM lesions**: MWoA = HC
22	Zhang et al., 2017	MWoA = HC		MWoA = HC	**GM**: MWoA > HC: bilateral cerebellar culmen (lobule I-IV and lobule V) extending to the lingual gyrus, thalamus, fusiform and parahippocampal gyrus **Cortical thickness**: MWoA > HC: left inferior temporal and lateral occipital cortex, MWoA < HC: right insula **Gyrification index**: MWoA > HC: left postcentral gyrus, superior parietal lobule and right lateral occipital cortex MWoA < HC: left rostral middle frontal gyrus
23	Szabo et al., 2018	MWA > HC: CC trend MWoA = HC MWA > MWOA: left parieto-occipital WM	MWA < HC: CC, bilateral parieto-occipital and cingulate WM MWoA = HC MWoA = MWA	**AD:** M = HC **RD:** MWA < HC: CC, bilateral parieto-occipital and cingulate WM MWoA = HC MWoA = MWA	Negative correlation between AD value of left SLF and left CST with disease duration in MWA Negative correlation AD right SLF and frequency of migraine attacks in MWA No significant correlation in MWOA
24	Marciszewski et al., 2018		M > HC: medullary raphe/SpV, left dlPons, left midbrain and PAG	M > HC: medullary raphe/SpV, left dlPons, left midbrain PAG	**GM**: M < HC in M medullary raphe/SpV, dorsomedial pons and bilateral dlPons MWoA = MWA
25	Petrušić et al., 2018	MWA = HC No significant difference was seen between MWA and HC	MWA = HC MWA = MWA +	**AD:** MWA < HC: trend in forceps minor, left UF, and right ATR MWA = HC MWA = MWA + MVA + < MVA: trend in forceps minor and right ILF **RD:** MWA < HC: trend in right cingulum cingulate tract No significant difference was seen between MWA and HC No significant difference was seen between MWA and MWA + groups MVA + < MVA: trend in right SLFT, SLFP, and CAB	Negative correlation between frequency of aura and mean AD/MD of the right ATR
26	Shibata et al., 2018	MWoAD + < HC: genu, body, splenium, and major/minor forceps of the CC, and the bilateral OR MWAD- < HC: genu, body, splenium, and major/minor forceps of the OR MWoAD + , MWAD- < HC: body of the CC	MWoAD + , MWoAD-, MWAD- > HC: genu, body, splenium, and major/minor forceps of the CC	**AD**: MWOAD + = MWOAD- = MWAD = HC **RD**: MWOAD + = MWOAD- = MWAD- > HC: genu, body, splenium, and major/minor forceps of the CC	No correlations between durations of disease, frequency of migraine attack and FA values of the CC
27	Chong et al., 2019		M > PPTH: left CAB M > HC: right CAB, bilateral CCG	M > PPTH: bilateral CAB, M < PPTH: right cingulum M > HC: right CAB, left UF, bilateral CCG (MQ1 and MQ3) M < HC: bilateral CCG (MQ2)	Positive association between migraine frequency and MD of forceps major
28	Marciszewski et al., 2019	M > HC: area surrounding the ventral trigeminothalamic tract/ventral tegmental area during all three phases FA in patients was not different during either phase	Interictal period: M > HC: left SpV, left dlPons, right dmPons/dlPons, midbrain PAG, cuneiform nucleus 24-h period before: M = HC: SpV, PAG/CNF, dlPons and dmPons regions 72-h after: M > HC: dm/dlPons and PAG/CNF	**AD**: M > HC: dlPons and dmPons during the interictal and after migraine phases **RD**: M = HC	MD and FA during the interictal phase were not significantly correlated to migraine frequency, years, and intensity of migraine pain
29	Kattem Husøy et al., 2019			**AD**: M > headache free group: CC, CST, IFOF, ILF and left SLF	After correcting for Fazekas, age, sex, anxiety/depression, chronic pain and consumption of alcohol and analgesics, increased AD was still observed in CC, right IFOF, right ILF and right CST in M compared to HCs No correlation between DTI indices and frequency of headache attacks
30	Qin et al., 2019	MWoA < HC: vermis VI extending to the bilateral lobule V and the bilateral lobule VI of the cerebellum	MWoA > HC: right inferior cerebellum peduncle, spinal trigeminal nucleus	MWoA > HC: right inferior cerebellum peduncle, spinal trigeminal nucleus	**GM volume**: MWoA < HC in SpV
31	Planchuelo-Gómez et al., 2020	EM = CM = HCs	EM = CM = HCs	**AD:** CM < EM: widespread findings **RD:** EM = CM = HCs	**Covariate analysis:** *Time from onset of CM*: CM < EM: Significant decreased AD values were found in the middle cerebellar peduncle, bilateral superior peduncle, left inferior cerebellar peduncle, left EC, pontine crossing tract and right CST) *duration of migraine*: no significant difference **Correlation**: Significant positive correlations between time from onset of CM and mean FA in the bilateral EC. Significant negative correlations between time from onset of CM and mean RD in the bilateral EC. No significant correlations were seen neither for mean MD or mean AD, nor for duration of migraine and migraine frequency
32	Russo et al., 2020	MWoA dCA < HC: CC MWoA dCA < MWoA ndCA: CC	MWoA dCA = HC MWoA dCA = MWoA ndCA	MWoA dCA = HC MWoA dCA = MWoA ndCA	No significant differences in clinical parameters of migraine (disease duration, pain intensity, MIDAS, headache impact test 6, and psychological assessment) between different migraine subgroups
33	Coppola et al., 2020	EMWoA = HCs CMWoA < EMWoA: bilateral SCR and PCR, bilateral body of the CC, right SLF, and right forceps minor	EMWoA = HC CMWoA > HC: right SCR and PCR, right SLF, and right splenium of the CC CMWoA vs. HC groups showed overlapping WM tracs, indicating an increase in MD in right PCR and right SCR CMWoA > EMWoA: bilateral SCR and right PCR, right body of the CC, right SLF, right splenium of the CC, and right PLIC	**AD**: EMWoA = HCs **RD**: EMWoA = HCs CMWoA > HC: bilateral SCR and PCR, bilateral genu of the CC, bilateral PLIC, and bilateral SLF	FA value of right PCR in EMWoA had negative correlation with the duration of migraine attacks In EMWoA, the MD value of the right PCR had a significant positive correlation with the severity of migraine headaches In the CMWoA, there was a tendency towards a negative association between FA value of the right PCR with the severity of migraine headaches
34	Masson et al., 2021a, b	M = HC	M = HC	M = HC	**WM volume:** M > HCs: left hemisphere, intersecting SLF and SCR, (superior temporal areas and the postcentral gyrus)
35	Pak et al., 2021	MOH + > MOH-: left OFC			Significant correlation between MOH and FA of the left OFC

*ACR* anterior corona radiata; *AD* axial diffusivity; *ADC* apparent diffusion coefficient; *ALIC* anterior limb of the internal capsule; *ATR* anterior thalamic radiation; *CAB* cingulum–angular bundle; *CC* corpus callosum; *CCG* cingulum cingulate gyrus; *CM* chronic migraine; *CMWoA* chronic migraine without aura; *CNF* cuneiform nucleus; *Complicated MWoA* MWoA patients with depressive/anxious disorder; *CR* corona radiate; *CSF* cerebrospinal fluid; *CST* corticospinal tract; *dCA* developing cutaneous allodynia; *dlPons* dorsolateral pons; *dmPons* dorsomedial pons; *DT* diffusion tensor; *DTI* diffusion tensor imaging; *EC* external capsule; *EM* episodic migraine; *EMWoA* episodic migraine without aura; *FA* fractional anisotropy; *GM* grey matter; *HCs* healthy controls; *HF* migraine with a high attack frequency (> 3 migraine attacks per month); *IC* internal capsule; *ILF* inferior longitudinal fasciculi; *IFOF* inferior fronto-occipital fasciculus; *LD* migraine patients with long disease duration (more than 15 years of migraine attacks); *LF* migraine with low attack frequency (< 3 attacks per month); *LTCM* patients with long-term chronic migraine; *M* migraine patients; *MD* mean diffusivity; *MIDAS* the migraine disability assessment; *MOH +*  Medication overuse headache; *MOH-* did not overuse medication; *MQ* mean quartile; *MT +*  middle temporal visual area; *MWA* migraine with aura; *MWoA* migraine without aura; *MWoAD +*  MWoA with medication overuse headache; *MWOAD-* MWoA without medication overuse headache; *MWAD +*  MWA with medication overuse headache; *MWAD-* MWA without medication overuse headache; *ndCA* non-developing cutaneous allodynia; *OFC* orbitofrontal cortex; *OR* optic radiation; *PAGs* periaquaductal gray matter; *PCR* posterior corona radiata; *PLICs* posterior limbs of internal capsules; *PPTH* persistent post-traumatic headache; *PTR* posterior thalamic radiation; *RD* radial diffusivity; *RIC* retro-lenticular internal capsule; *ROI* region of interest; *SCR* superior corona radiate; *SD* migraine patients with short disease duration (< 15 years of migraine attacks); *SDS +*  self-rating depression scale > 49; *SDS-* self-rating depression scale <  = 49; Simple *MWoA* MWOA patients without depressive/anxious disorder; *SLF* superior longitudinal fasciculi; *SpV* spinal trigeminal nucleus; *SWM* subcortical white matter; *TBSS* tract-based spatial statistics; *UF* uncinate fasciculus; *V3A* one of visual cortical areas; *WM* white matter. = marks no significant differences

CC was one of the major tracts showing significant differences in migraine. Decreased FA (Russo et al., 2020; Yu et al., 2013a, b; Yuan et al., 2012), MD (Chong et al., 2019; Messina et al., 2015; Szabo et al., 2018; Yu et al., 2013a, b), AD (Messina et al., 2015; Petrušić et al., 2018; Yu et al., 2013a, b) and RD (Chong et al., 2019; Messina et al., 2015; Petrušić et al., 2018; Szabo et al., 2018) values were observed particularly in the genu, body and splenium of the CC of migraine patients compared to HCs. Decreased FA (Yu et al., 2013a, b), MD (Chong et al., 2019; Messina et al., 2015; Szabo et al., 2018), AD (Messina et al., 2015), and RD (Chong et al., 2019; Messina et al., 2015; Szabo et al., 2018) in cingulate fibers was also detected. Compared to HCs, lower FA in the brainstem tracts (Schmitz et al., 2008; Yu et al., 2013b) and CST (Yu et al., 2013a), and higher MD, AD, and RD in brainstem tracts, including medullary raphe/SpV, dorsolateral and dorsomedial pons, midbrain PAG and cuneiform nucleus (CNF), were reported (Marciszewski & Meylakh, 2019; Marciszewski et al., 2018). Besides, migraine pediatric cohorts showed lower RD values in the trigeminothalamic tract (Messina et al., 2015).

Several studies included in this systematic review observed decreased MD, AD, and RD in the thalamus and thalamic radiations in patients compared to HCs (Chong et al., 2019; Chong & Schwedt, 2015; Coppola et al., 2014; Messina et al., 2015; Petrušić et al., 2018; Yu et al., 2013a, b). Similarly, pediatric patients showed lower MD, AD, and RD in WM tracts that connected either the thalamus to the cortex or from sensory nerves to the thalamus, including trigeminothalamic and thalamocortical pathways (Messina et al., 2015). However, FA alterations of the thalamus varied during different migraine attack phases. Microstructural changes in association fiber tracts were also reported in patients with migraine headaches. Several studies observed lower MD and AD in the left SLF fibers in migraine patients compared to HCs, along with both lower and higher RD (Kattem Husoy et al., 2019; Messina et al., 2015; Yu et al., 2013a, b). Besides, both increased and decreased MD (Chong & Schwedt, 2015; Messina et al., 2015), AD (Kattem Husoy et al., 2019; Messina et al., 2015), and RD (Chong & Schwedt, 2015; Messina et al., 2015) values were reported in the inferior longitudinal fasciculus (ILF) tract of migraine patients without any significant FA change. In the uncinate fasciculus, reduced left AD and increased bilateral MD were observed (Chong et al., 2019; Petrušić et al., 2018). Gomez-Beldarrain et al. after a three-months follow-up reported no significant between-group differences in the uncinate fasciculus microstructure. However, migraine patients who presented migraine attacks after six months from the treatment (referred as long-term chronic migraine), showed lower FA in this structure, as well as in the right anterior insula, and the bilateral cingulate (Gomez-Beldarrain et al., 2015). Finally, only two studies reported reduced FA in the cerebellum (Qin et al., 2019; Schmitz et al., 2008). Higher MD, AD, and RD values were also detected in the right inferior cerebellar peduncle (Qin et al., 2019).

### Diffusion metrics changes during migraine attack phases

Several studies assessed microstructural changes of thalami in different phases of the migraine cycle, reporting FA reduction mainly in MWoA patients (DaSilva et al., 2007; Yu et al., 2013a, b). By contrast, Coppola et al., reported higher FA in bilateral thalami in comparison to HCs (Coppola et al., 2016b; Coppola et al., 2014). Notably, thalamus FA and MD values normalized during an attack and thalamic FA showed a positive correlation with the number of days lasted from the latest attack (Coppola et al., 2016a; Coppola et al., 2014). Marciszewski et al. (2018) investigated brainstem pain processing regions alterations across the migraine cycle reporting higher MD and AD in several brain areas, including the SpV, dorsolateral and dorsomedial pons, PAG and CNF regions of the midbrain during the interictal phase. MD values returned to normal levels from the 24-h period prior to the migraine attack. Then, MD and AD increased again in the dorsomedial and dorsolateral pons and PAG/CNF, within the 72-h period after the migraine attack. Similarly, migraine patients showed higher RD values in the SpV, and PAG/CNF during the interictal period, while no significant differences were observed immediately after a migraine attack. On the other hand, FA increased in the medial lemniscus/ventral trigeminal thalamic tract over the entire migraine cycle (Marciszewski & Meylakh, 2019).

### Migraine with aura vs. migraine without aura

Eight studies investigated differences in WM microstructures between these migraine subgroups, three of which reported significant WM changes. Significant lower FA of the right OR and the bilateral ventral trigeminothalamic tract in MWA (Rocca et al., 2008) and lower FA of the ventrolateral PAG in MWoA patients (DaSilva et al., 2007) were observed. In addition, it has been reported increased FA of the left parieto-occipital WM in MWA compared to MWoA patients (Szabo et al., 2018). Similarly, Qin et al. (2019) found that MWoA patients had higher MD, AD, and RD in the spinal trigeminal nucleus compared to HCs (Qin et al., 2019). By contrast, several studies reported no significant difference between these two groups in FA (Chong & Schwedt, 2015; Granziera et al., 2006; Messina et al., 2015; Tedeschi et al., 2016; Tessitore et al., 2015), MD (Chong & Schwedt, 2015; Messina et al., 2015; Szabo et al., 2018; Tedeschi et al., 2016; Tessitore et al., 2015), AD (Messina et al., 2015; Tedeschi et al., 2016; Tessitore et al., 2015) and RD (Chong & Schwedt, 2015; Messina et al., 2015; Szabo et al., 2018; Tedeschi et al., 2016; Tessitore et al., 2015). Migraine patients who experienced additional somatosensory and dysphasic symptoms besides visual aura (MVA +) displayed both lower MD in the cingulum angular bundle and the right SLF and lower RD in the former structure compared to patients with only visual aura (MVA), although the difference was non-significant (Petrušić et al., 2018).

Brain diffusion differences in MWA with medication overuse headache (MOH), MWoA with MOH and MWoA without MOH compared to HCs has also been investigated. MWoA with MOH and MWA without MOH groups showed a considerable FA decrease in the CC compared to HCs, while MWoA without MOH group showed no significant difference. Both MD and RD in the CC were significantly higher in all the three patient subgroups, while AD showed similar results between HCs and patient subgroups (Shibata et al., 2018).

### Chronic vs. episodic migraine

Four studies investigated microstructural alterations in CM (Coppola et al., 2020; Gomez-Beldarrain et al., 2015; Neeb et al., 2015; Planchuelo-Gómez et al., 2020), reporting microstructural alterations in both CM and EM patients compared to HCs. CM compared to EM patients showed lower FA value in the bilateral superior and posterior corona radiata, PLIC, SLF, and CC (the body and splenium parts) (Coppola et al., 2020). Planchuelo-Gómez et al. after adjusting for time onset, identified lower AD values in multiple regions in CM compared to EM (superior, middle, and inferior cerebellar peduncle, left EC, pontine crossing tract). No significant differences were seen after correcting for total disease duration and for the presence of aura (Planchuelo-Gómez et al., 2020). In addition, no significant differences were detected between CM patients and HCs (Planchuelo-Gómez et al., 2020). Notably, EM patients showed comparable DTI indices with HCs (Coppola et al., 2020; Neeb et al., 2015; Planchuelo-Gómez et al., 2020). Gomez-Beldarrain et al. followed migraine patients for six months aimed at identifying persistent chronic migraine. They performed a ROI-based study focusing to the insula, cingulate gyrus, and uncinate fasciculus. Diffusion imaging after three months revealed no FA value differences in the aforementioned ROI between HC, EM, and CM. At six months, only nine CM patients out of 18 were still classified as CM showing lower insula, cingulate gyri and uncinate fasciculus FA (Gomez-Beldarrain et al., 2015).

### Migraine with comorbid depression/anxiety

WM integrity in migraine patients associated with depressive or anxiety symptoms was evaluated in three studies (Gomez-Beldarrain et al., 2015; Li et al., 2011; Yu et al., 2013a, b). MWoA patients were divided into two groups according to the self-rating depression scale (SDS), one with severe depressive symptoms (SDS score > 49; SDS + group) and one with low depressive symptoms (SDS score < 49; SDS- group). The SDS- group compared to the control group showed reduced MD, AD, and RD in widespread WM tracts, including CC, corona radiata, thalamic radiations, internal and external capsules, and SLF, while no differences were reported for FA. By contrast, SDS + patients, compared to HCs, exhibited reduced FA, along with reduced AD, and increased RD in WM tracts including CC, corona radiata, internal and external capsule, and cerebral peduncle, despite no difference in MD. Moreover, SDS + patients compared to their SDS- counterpart showed reduced FA, increased MD, and increased RD in the CC, corona radiata, internal and external capsules, and SLF, while no differences were reported for AD. Correlation analysis showed significant negative correlations between SDS scores and mean FA of the anterior corona radiata, CC, and SLF. Besides, significant positive correlations between SDS and the mean RD of CC (genu and splenium) and SLF were observed (Yu et al., 2013a, b). Moreover, in the study by Li et al. (2011), CC integrity in MWoA patients without depressive/anxiety symptoms was compared with MWoA patients showing these symptoms. Decreased FA was reported in both migraine groups compared to HCs, in line with the literature previously reported. Patients with depressive/anxiety symptoms showed lower mean FA compared to those without these symptoms. In addition, FA value of the CC showed a significant negative correlation with anxiety/depression scores (Li et al., 2011). Interestingly, lower FA in patients with higher anxiety symptoms was replicated by Gomez-Beldarrain et al. (Gomez-Beldarrain et al., 2015).

### Correlations with demographic and clinical features

No significant correlations were observed between patients' age and DTI indices (Coppola et al., 2014; Kara et al., 2013; Li et al., 2011)). Similarly, several studies reported no significant correlation between disease duration and frequency of migraine attacks with DTI measurements (Russo et al., 2020; Shibata et al., 2018) (DaSilva et al., 2007) (Coppola et al., 2014; Kara et al., 2013); (Szabo et al., 2012); (Coppola et al., 2020; Marciszewski et al., 2018; Rocca et al., 2008). Accordingly, DTI parameters showed no correlation with disease duration and attack frequency in pediatric migraine patients (Messina et al., 2015). However, it has been suggested a positive association between disease duration and MD of the ATR and CST (Chong & Schwedt, 2015) and a negative relationship with FA of the CC (Yu et al., 2013a; Yuan et al., 2012), PLIC (Yu et al., 2013a), and both MD and AD of the CC, and the anterior/posterior limb of the internal capsule (Yu et al., 2013a). In MWA patients, CST and SLF AD values were negatively correlated with disease duration (Szabo et al., 2018). Significant negative correlations were observed between the frequency of migraine attacks and mean FA, MD, and AD of the CC and PLIC (Yu et al., 2013a). Similarly, a positive association between MD of the CC major forceps with the frequency of migraine attacks was reported (Chong et al., 2019). Additionally, aura frequency in MWA was negatively associated with AD and MD of the ATR (Petrušić et al., 2018).

In CM patients, negative correlations were observed between attack frequency and disease duration with FA of the CC (Li et al., 2011). However, onset time of CM showed positive correlations with FA of the EC (Planchuelo-Gómez et al., 2020). A negative correlation was also observed between CM onset and mean RD of the EC (Planchuelo-Gómez et al., 2020). Coppola et al. (Coppola et al., 2020) revealed a trend toward negative and positive correlations between pain intensity and FA of the posterior corona radiata in CM and EM patients, respectively(Coppola et al., 2020)(Coppola et al., 2020). However, different independent studies did not report significant associations between pain intensity and brain diffusion metrics (Coppola et al., 2014; Li et al., 2011; Marciszewski et al., 2018; Russo et al., 2020).

## Discussion

In this article, we reviewed brain microstructural alterations in patients with migraine headaches. In summary, the migraine DTI literature showed heterogeneous findings, mainly in the comparison between migraine patients and HCs. Furthermore, no consistent evidence was reported regarding WM differences between MWA and MWoA and in chronic migraine patients, while differences during different phases of migraine attacks were prominent.

### Migraine-associated changes in WM microstructure

Regarding studies reporting migraine-associated changes in WM microstructure (mainly decreased FA and increased MD, suggesting decreased WM integrity), thalamus, CC, SLF, ILF, cingulum, OR, and CR showed the highest vulnerability. Structural changes in these brain regions might be linked with different mechanisms, such as maladaptive neural plastic changes, reduced myelination of neuronal sheath and neuronal degeneration (Li et al., 2011; Szabo et al., 2012), or changes secondary to chronic pain (Li et al., 2011; Messina et al., 2015). The thalamus is a key GM structure functioning as a center for relaying sensory and motor information, and the ATRs are brain structures involved in modulating pain (Chong & Schwedt, 2015). In most of the studies, the migraine-associated microstructural changes in the thalamus were dependent on the time of imaging according to the phases of migraine cycle.

Similarly, CC, a large-sized WM structure that interconnects both cerebral hemispheres, contributes to the pain processing system by regulating pain control (Li et al., 2011; Yuan et al., 2012) besides transmission and integration of information (Rotarska-Jagiela et al., 2008). Generally, decreased FA, MD and RD were reported in different parts of CC. These findings can mostly be attributed to axonal loss.

Cell death, demyelinating processes, intra/extracellular water changes resulting from repetitive pain stimulations can manifest as decreased FA and increased MD. However, due to possible confounding factors that affect DTI indices, such as partial volume effect, standard streamline tractography, WM tract subtypes, and crossing fibers, such as the CC (O'Donnell & Westin, 2011), caution is needed in interpreting the results. Thus, inconsistencies of findings, in addition to a recent meta-analysis in migraine patients reporting no differences in GM/WM morphology (Masson et al., 2021a, b), might suggest that microstructural brain changes in migraine are not intrinsic to the disease and several factors might influence brain microstructures in migraine.

### Diffusion metrics changes during migraine attack phases

Decreased AD, RD and MD indices in the thalamus and ATR was observed in migraine patients between attacks except by Coppola et al. reporting increased FA in the interictal phase (Coppola et al., 2016b; Coppola et al., 2014). Interestingly, it was reported that thalamic FA and MD metrics were not significantly different from HCs during migraine attacks (Coppola et al., 2016a; Coppola et al., 2014). Increased FA during the interictal phase might be attributed to reduced dendritic branching and neuronal connections, concurrent with intact cell density between the attacks (Coppola et al., 2014). Furthermore, during the attack, the number of dendritic branches increased (Coppola et al., 2016a; Coppola et al., 2014). This result may be interpreted, at least partly, within the habituation deficit framework. Besides, decreased activity of thalamocortical connections (i.e., habituation) vanished 12 h before or after migraine attack onset and presented a pattern similar to controls, which might be interpreted as a thalamic pre-activation plastic neuronal changes in the thalamus following increased activity of serotonergic afferent pathways (Coppola et al., 2005, 2010, 2014; DaSilva et al., 2007; de Tommaso et al., 2005; Judit et al., 2000; Stankewitz et al., 2013).

In addition, diffusivity markers (MD, AD, and RD) were generally increased during the interictal phase and immediately after migraine attacks in the brainstem (Marciszewski & Meylakh, 2019). MD values returned to normal levels in these regions immediately before an attack. Meanwhile, FA value of the medial lemniscus/ventral trigeminal thalamic tract increased in all the three stages and, unlike the other indices, remained high before the migraine attack. Although findings were partially contrasting, these studies suggest that further analyses aimed at assessing fluctuations of microstructural properties during the different stages of migraine are needed to fully characterize the pathophysiology of this disorder (Marciszewski & Meylakh, 2019).

### Microstructural alterations in different migraine subtypes

Several studies explored brain differences in migraine subtypes through different imaging techniques. For instance, the visual cortex showed stronger activity patterns in response to visual stimulation in MWA compared to MWoA patients, as measured via functional MRI (Cucchiara et al., 2015; Datta et al., 2013; Kincses et al., 2019). This effect was coupled with greater functional connectivity during resting-state, suggesting consistent alterations in the brain functional organization between migraine phenotypes. (Faragó et al., 2017; Tedeschi et al., 2016). By contrast, few studies reported significant differences in brain microstructural features between these subgroups, while most of the studies included in the present review revealed no significant difference. These results might suggest that migraine subgroups are not microstructurally different, or that the potential effect cannot be detected by diffusion analysis. Additionally, several confounding factors might influence the detection of significant differences, leaving open the question whether different migraine subgroups are linked with specific microstructural features.

Despite the inconsistency of findings comparing CM and EM or CM and HCs, non-significant differences in WM microstructure between EM and HCs were consistently reported, suggesting that the mechanisms underlying WM microstructural changes in migraine might be dependent, at least partly, on its chronicity (Coppola et al., 2020; Neeb et al., 2015; Planchuelo-Gómez et al., 2020). Interestingly, prior studies reported a decrease in the number of streamlines within the temporal lobe in patients with CM compared to EM (Planchuelo-Gomez & Garcia-Azorin, 2019). In contrast, CM patients showed an increase in the number of streamlines in several subcortical regions, such as the caudate, thalamus, hippocampus, and in the superior frontal gyrus (Li et al., 2017; Planchuelo-Gomez & Garcia-Azorin, 2019), suggesting potential compensatory mechanisms aimed at counteracting axonal loss through neuronal plasticity. Further studies are required to confirm or rule out the effects of chronic migraine on WM integrity and to assess the multiple factors linked with the high heterogeneity of the results.

### Association of psychiatric symptoms and diffusion metrics

Alteration of diffusivity indices in the CC parts (genu, body, and splenium), as well as the internal capsule (IC) and SLF, were reported in migraine patients with depressive/anxiety symptoms compared to their counterparts without symptoms. Similarly, higher severity of depressive symptoms was related to greater WM disintegration in these fiber tracts (Cole et al., 2012). These fiber tracts have shown microstructural alterations in patients with major depressive disorder, suggesting that microstructural changes observed in migraine patients with depressive symptoms could be due to either of these entities (Korgaonkar et al., 2011; Li et al., 2011; Mettenburg et al., 2012; Yu et al., 2013b; Zhang et al., 2012). Comparing studies which investigated depression and migraine as separate entities and studies focused on concomitant migraine and depression could be helpful in unraveling the distinction between brain diffusivity alterations inherent to these disorders. As decreased AD in WM tracts such as CC, IC and EC was reported in both depressed and non-depressed patients with migraine, axonal loss might be attributable to migraine pathophysiology rather than depressive symptoms, while RD increase and FA reduction, which are presumed markers of demyelination, are observable more frequently in MWOA patients with depressive symptoms (van Velzen et al., 2020; Yu et al., 2013b). Thus, it can be assumed that axonal degeneration and brain atrophy might highlight the adaptive reaction of neurons in response to frequent migraine attacks, while demyelination might represent the main response to depressive symptoms (Li et al., 2011). Future studies should confirm these assumptions.

### Correlation between clinical variables and DTI indices in migraine

Some of the studies discussed in the present review assessed the correlation between age and DTI indices with contrasting results. It is worthy of note that participants of the included studies were mainly middle-aged, a period of time when microstructural changes are minimal (Behler et al., 2021). For the aim of understanding age-related microstructural changes in migraine, DTI metrics should be carefully evaluated through longitudinal studies. Similarly, no correlations were reported between the main DTI indices with disease duration, frequency of migraine attacks, and pain intensity. However, preliminary evidence reported a significant positive association between both attack frequency and disease duration with higher MD and lower FA mainly within the CC, thalamic radiations, and CST. These findings, although inconsistent and heterogeneous, might suggest potential deleterious effects of frequent migraine attacks on myelination, which might emerge as a function of the severity of the disease. This might explain the heterogeneity of the findings, although further studies should assess this relationship to unravel the underlying pathophysiological mechanisms influencing brain microstructure by migraine attacks.

### Beyond diffusion tensor imaging

Diffusion MRI investigate microstructural structures *in vivo* in the biologic tissue, detecting early WM microstructural changes. However, to date, DTI indices are non-specific biomarkers for several neurological disorders, including migraine (Alexander et al., 2007). The lack of specificity might depend on some limitations, including subject motion, image resolution, partial volume effect, and crossing WM fibers resulting in potentially biased FA computation (Alexander et al., 2007; Pasternak et al., 2018). Furthermore, DTI technique owns several intrinsic limitations in the detection of GM abnormalities. Indeed, FA can detect water diffusion restriction in anisotropic areas, while GM, consisting of neuronal body, has isotropic properties as diffusion of water molecules is not restricted (Ghazi Sherbaf et al., 2018). These issues might confound the results of migraine studies, masking some potential effects.

Additionally, numerous WM brain structures consist of several complex fiber arrangements (e.g., the CR and the WM adjacent to the cortex), and due to the multiple cross-fibers, DTI indices in these tracts might be suboptimal (Deligianni et al., 2016; Pasternak et al., 2018). Moreover, in neurological disorders, brain microstructure can be affected by the combination of several alterations such as gliosis, inflammation, demyelination, axonal loss and plastic neuronal changes, which are known to affect DTI metrics, making the interpretation of such alterations problematic (Alexander et al., 2007; Pasternak et al., 2018). Moreover, DTI model assumes Gaussian distribution for diffusion within each voxel (Pasternak et al., 2018; Winston, 2015), which is not necessarily the case at the whole brain level (Ghazi Sherbaf et al., 2018). These limitations highlight the necessity to apply novel advanced modalities in migraine, such as Diffusion kurtosis imaging (DKI) and neurite orientation dispersion and density imaging (NODDI). These imaging methods might improve the sensitivity, detecting subtle alterations. DKI is based on non-Gaussian diffusion (Ito et al., 2016), which might improve the detection of diffusion abnormalities in both isotropic (e.g., GM) and anisotropic regions based on the degree of diffusion restriction (Ghazi Sherbaf et al., 2018). DKI consists of three main kurtosis indices, including mean kurtosis (MK), axial kurtosis (AK), and radial kurtosis (RK), representing the structural complexity. Overall, these parameters showed higher sensitivity for detecting crossing fibers compared to DTI and are less prone to the partial volume effect and CSF contamination.

NODDI is a novel model to detect morphology of neurites. Similar to DKI, the model is more suitable for both GM and WM, and less CSF contamination is expected for this modality. Nevertheless, NODDI can be applied to diffusion MRI data typically acquired in clinical setting, which might hasten its extensive application in the clinical research field (Winston, 2015). Other techniques might provide valuable microstructural information in migraine, such as the diffusion ensemble average propagator (EAP), although this methodology requires multi-shell acquisitions (and longer acquisition time), making it less feasible within the clinical practice. This limitation can be overcome by applying novel approaches. Apparent Measures Using Reduced Acquisitions (AMURA), a technique assuming that diffusion anisotropy is approximately independent of the radial direction (Aja-Fernández et al., 2020), could reduce the acquisition time. This approach has been recently applied in migraine patients, showing promising results to detect microstructural changes associated with this disorder (Planchuelo-Gómez et al., 2020). Further studies should evaluate the application of advanced statistical and machine learning techniques aimed at assessing the latent relationships between migraine features and microstructural changes, such as multimodal canonical correlation analysis combined with joint independent component analysis (Planchuelo-Gómez et al., 2021).

### Limitations and future direction

Despite its strengths, this systematic review is prone to some limitations. About 20% of the included studies were drawn from similar, or partially overlapping, samples of participants, which might have influenced some results. Moreover, despite our efforts to discuss both significant and non-significant results, there is a tendency towards publication of papers with significant results compared to non-significant ones (i.e., publication bias). Indeed, most of the studies reported significant changes in at least one diffusion parameter, while only nine studies reported completely non-significant results among the investigated DTI parameters. Furthermore, the studies included in this review might be prone to some intrinsic limitations, which should be addressed by future studies. First, the number of patients included in most of these studies was lower than 50, which might limit the generalizability of the conclusion that can be drawn. Additionally, as reported in the present review, imaging time at different phases through migraine cycle might influence the results, due to the fluctuations of DTI parameters in the different phases of migraine. Some possible confounding factors (e.g., positive family history of migraine, severity of pain, presence or absence of aura, types of auras, and anxiety/depression profiles of participants, patient's age and sex, medication intake) might influence DTI findings. Similarly, heterogeneity in the study design, clinical features, and analysis methods might lead to conflicting results, making the interpretation of microstructural alterations in migraine more complex (Forkel et al., 2020). Depression also has an effect on brain microstructure, especially the genu of the CC and the left ALIC (Chen et al., 2016). Therefore, it should be considered for patient selection or classification. Moreover, no study has assessed whether a positive family history of migraine or a genetic predisposition is linked to WM microstructure alterations. Finally, most of the studies were cross-sectional, thus it is impossible to infer a cause-effect relationship between migraine and DTI. Longitudinal studies will be extremely helpful to investigate the development of microstructural abnormalities during disease or treatment.

## Conclusion

Despite the great effort to investigate the pathophysiology of migraine, the evidence summarized here suggests that future studies are still necessary to unravel brain diffusion alterations linked with this disorder. Preliminary evidence suggest that microstructural alterations occur during the disease. Reduced microstructural integrity was observed in the thalamus, CC, longitudinal fasciculus, and cingulum in patients with migraine compared to controls. However, the tensor model was unable to find remarkable differences between different migraine subtypes. Notably, changes in DTI indices occur in the interictal phase, which might be interpreted within the habituation deficit theory or as neuronal plasticity mechanisms. Moreover, these results might suggest that frequent stimulation and CSD events lead to release of neurotransmitters and pain generation, and finally, cellular damage, as captured by DTI indices. Indeed, repetitive occurrence of neuronal damage can be associated with disruption of WM microstructure and decreased FA in several brain microstructures. In chronic migraine, the variable equilibrium between neuronal damage, due to repetitive pain stimulations, and plastic neuronal changes occurring as compensatory mechanisms might lead to higher heterogeneous results. DTI assessment and interpretation of structural abnormalities in migraine are still questionable due to the complexity of migraine pathophysiology and DTI limitations. Further longitudinal studies, applying novel advanced modalities are required to fully understating the effects of migraine on brain structural connectivity and its progression.

## Data Availability

Not applicable.

## Abbreviations

MOH: Medication overuse headache
MWA: Migraine with aura
MWoA: Migraine without aura
DTI: Diffusion tensor imaging
FA: Fractional anisotropy
MD: Mean diffusivity
AD: Axial diffusivity
RD: Radial diffusivity
PAG: Periaqueductal gray matter
GM: Gray matter
WM: White matter
ILF: Inferior longitudinal fasciculus
SLF: Superior longitudinal fasciculus
UF: Uncinate fasciculus
CC: Corpus callosum
CSD: Cortical spreading depression
PLIC: Posterior limb of internal capsule
ROI: Region of interest
ACR: Anterior corona radiata
PCR: Posterior corona radiata
PTR: Posterior thalamic radiation
ATR: Anterior thalamic radiation
IC: Internal capsule
EC: External capsule
SpV: Spinal trigeminal nucleus
CST: Corticospinal tract
CFN: Cuneiform nucleus
SDS: Self-rating depression scale
CM: Chronic migraine
HC: Healthy control
